# Peptidylarginine Deiminase Isozyme-Specific PAD2, PAD3 and PAD4 Inhibitors Differentially Modulate Extracellular Vesicle Signatures and Cell Invasion in Two Glioblastoma Multiforme Cell Lines

**DOI:** 10.3390/ijms21041495

**Published:** 2020-02-22

**Authors:** Pinar Uysal-Onganer, Amy MacLatchy, Rayan Mahmoud, Igor Kraev, Paul R. Thompson, Jameel M. Inal, Sigrun Lange

**Affiliations:** 1 Cancer Research Group, School of Life Sciences, University of Westminster, London W1W 6UW, UK; P.onganer@westminster.ac.uk; 2 School of Life Sciences, University of Westminster, London W1W 6UW, UK; amy.maclatchy@my.westminster.ac.uk (A.M.); w1700237@my.westminster.ac.uk (R.M.); 3 Electron Microscopy Suite, Faculty of Science, Technology, Engineering and Mathematics, Open University, Milton Keynes MK7 6AA, UK; igor.kraev@open.ac.uk; 4 Department of Biochemistry and Molecular Pharmacology, University of Massachusetts Medical School, Worcester, MA 01655, USA; paul.thompson@umassmed.edu; 5 School of Life and Medical Sciences, University of Hertfordshire, Hatfield AL10 9AB, UK; j.inal@herts.ac.uk; 6 School of Human Sciences, London Metropolitan University, London N7 8DB, UK; 7 Tissue Architecture and Regeneration Research Group, School of Life Sciences, University of Westminster, London W1W 6UW, UK

**Keywords:** peptidylarginine deiminases (PADs), protein deimination, extracellular vesicles (EVs), glioblastoma multiforme (GBM), prohibitin (PHB), Stromal interaction molecule 1 (STIM-1), moesin, microRNA (miR21, miR126, miR210), HIF-1

## Abstract

Glioblastoma multiforme (GBM) is an aggressive adult brain tumour with poor prognosis. Roles for peptidylarginine deiminases (PADs) in GBM have recently been highlighted. Here, two GBM cell lines were treated with PAD2, PAD3 and PAD4 isozyme-specific inhibitors. Effects were assessed on extracellular vesicle (EV) signatures, including EV-microRNA cargo (miR21, miR126 and miR210), and on changes in cellular protein expression relevant for mitochondrial housekeeping (prohibitin (PHB)) and cancer progression (stromal interaction molecule 1 (STIM-1) and moesin), as well as assessing cell invasion. Overall, GBM cell-line specific differences for the three PAD isozyme-specific inhibitors were observed on modulation of EV-signatures, PHB, STIM-1 and moesin protein levels, as well as on cell invasion. The PAD3 inhibitor was most effective in modulating EVs to anti-oncogenic signatures (reduced miR21 and miR210, and elevated miR126), to reduce cell invasion and to modulate protein expression of pro-GBM proteins in LN229 cells, while the PAD2 and PAD4 inhibitors were more effective in LN18 cells. Furthermore, Kyoto Encyclopedia of Genes and Genomes (KEGG) pathways for deiminated proteins relating to cancer, metabolism and inflammation differed between the two GBM cell lines. Our findings highlight roles for the different PAD isozymes in the heterogeneity of GBM tumours and the potential for tailored PAD-isozyme specific treatment.

## 1. Introduction

Peptidylarginine deiminases (PADs) are calcium-dependent enzymes which cause structural changes in target proteins via post-translational deimination, which affects protein function, protein–protein interactions, gene regulation and causes generation of neo-epitopes [1,2,3]. Protein deimination can also facilitate protein moonlighting, allowing proteins to exhibit a range of physiological and pathophysiological functions within one polypeptide chain [4,5]. PADs play important roles in cancer pathogenesis, including in the central nervous system [6,7,8,9]. Glioblastoma multiforme (GBM) is the most common and aggressive form of primary malignant brain tumour in adults. Standard treatment consists of surgical resection and radiotherapy in combination with temozolomide (TMZ) chemotherapy [10,11]. GBM has poor prognosis with only 28.4% of patients surviving one year and 3.4% surviving to year five [12,13,14]. Brain cancer cells are implicated in manipulating not only the tumour microenvironment but also systemic immunity to their advantage [15,16]. 

Extracellular vesicles (EVs) are recognised players in mediating such changes, being lipid bilayer-enclosed structures, 30–1000 nm in diameter, released from cells and acting as key-mediators for intra/inter-tumour communication through horizontal transfer of functional proteins and nucleic acids (mRNA, miRNA, lncRNA, sncRNA) [17,18,19,20]. EVs have great potential as diagnostic and prognostic biomarkers in a range of pathologies, including GBM, and therefore increased understanding of EV-mediated functions in GBM biology is urgently needed [21,22]. In GBM, EV protein-cargo has for example been associated with the phenotypic signature of GBM cells [23], while TMZ treatment has been shown to increase EV release in GBM and to promote more pro-oncogenic EV signatures [24]. Similarly, as in other cancers, GBM cells use EVs for inter-cellular communication in the tumour and to influence the surrounding microenvironment to promote tumour growth, angiogenesis, metabolism and invasion [20,25,26,27,28,29,30]. The regulation of EV biogenesis has therefore received increasing attention in recent years as an interceptive strategy in cancer, both to sensitize cancer cells to chemotherapy and to limit tumour growth in vivo [8,9,31,32,33,34,35,36,37,38]. The peptidylarginine deiminase (PAD)-mediated pathway of EV biogenesis has been highlighted as a novel and significant contributor to EV release in a range of cancer cells [8,9,33,36], including most recently in GBM by our group [9]. We previously showed that pan-PAD inhibition, using Cl-amidine, significantly modulated EV signatures, both with respect to EV numbers released, as well as by changing EV-related microRNA cargo to a more anti-oncogenic signature [9]. As Cl-amdine showed different effects on LN18 and LN229 GBM cells and due to the fact that it is a pan-PAD inhibitor with inhibitory effects on PAD2, PAD3 and PAD4 isozymes [39], further studies on assessing PAD2, PAD3 and PAD4 isozyme-specific inhibitors in these two GBM cell lines are warranted. Novel interventions for targeted EV regulation and inhibition may be of particular importance in GBM treatment, considering recent reports on TMZ-mediated increase in EV release and modulated EV cargos dedicated to cell adhesion processes therefore possibly increasing pro-tumoral communication by standard TMZ treatment [24]. 

Besides effects on EV release, PADs are well known for their contribution to a range of pathologies, including cancers, by converting arginines into citrulline, causing post-translational deimination and changes in target protein tertiary structure and function [1,2,3,8,40]. PADs are gaining increased attention in the context of GBM research. Previous studies have established that grade IV GBM patient samples show an increase in cytoplasmic and nuclear deiminated proteins, albeit not identifying specific protein candidates [7]. A recent study by our group established that the pan-PAD inhibitor Cl-amidine reduced deimination of the mitochondrial house-keeping protein prohibitin (PHB) and deimination of histone H3, as well as identifying a range of protein candidates that are deiminated in LN18 and LN229 cells under normal growth conditions [9]. This included prohibitin (PHB), a multifaceted protein with key roles in mitochondrial housekeeping and tumorigenesis [41,42,43,44]; Stromal interaction molecule 1 (STIM-1) which is a membrane ER-resident protein with important roles in calcium-homeostasis and cancer invasion [45,46,47]; and moesin, a critical factor for cell migration, filopodia formation [48] and associated with more aggressive forms of GBM [49,50]. The assessment of proteins involved in mitochondrial function, cancer progression and invasion is of considerable relevance both with respect to PAD-inhibitor mediated changes in total protein levels and with respect to their post-translational deimination, as this may affect protein structure, function and protein–protein interactions [1,40]. 

As five isozyme-specific PADs are known in mammals [1], which display tissue specific expression and different preferences for target proteins, the difference in prominence of the main three isozymes related to cancer and the CNS (PAD2,3 and 4) is of additional interest for isozyme-specific targeting relating to cancer types and cancer sub-types, including heterogeneous cancers like GBM. Indeed, in LN18 and LN229 GBM cell lines these three PAD isozymes have been found to be differently expressed [9] (Supplementary Figure S1A). An increase in PAD4 staining has been reported in undescribed astrocytomas [51], while PAD2 and PAD3 upregulation via cAMP-PKA signalling has been shown in U251MG astrocytoma cells [52]. This indicates some differences in PAD isozyme expression in different glioma and astrocytoma cells. In addition, PAD-upregulation was shown in a study assessing response to hypoxia in malignant gliomas [53]. 

Hypoxia related pathways may be of considerable relevance due to the hypoxic core of a tumour mass, which also contains the therapy resistant glioma stem-like cells, a well-recognized problem in the standard treatment of GBM tumours [10,11]. PAD activation has indeed been linked to hypoxia in the CNS [53,54,55] and deiminated KEGG (Kyoto Encyclopedia of Genes and Genomes) protein pathways for HIF-1 regulation have been identified to be enriched in animal models of hypoxia- and cancer-resistance [56,57]. PADs also modulate neuronal stem cell growth and death [58], which is of importance considering that GBM tumour invasion, progression and chemo-resistance of recurrent GBM are partly linked to stem-ness [59,60]. Therefore, effects of PAD inhibitors on PAD mediated pathways including EV-mediated export of a range of cancer and hypoxia related microRNAs are also of considerable interest. As we have previously shown pan-PAD inhibition to be effective for regulating EV release and two key microRNAs (miR21 and miR126) in GBM, we furthermore set out to assess the effects of PAD isozyme-specific inhibitors on these two miRs, as well as on the hypoxia-related miR210, which is related to more aggressive forms and poor prognosis in GBM [61,62,63]. Effects of the PAD-isozyme specific inhibitors on the invasion ability of GBM cells was also assessed, revealing isozyme-specific differences in the two different GBM cell lines, which also related to the effects observed on EV release inhibition. Furthermore, the PAD2, 3 and 4 isozyme-specific inhibitors differentially reduced total protein levels of PHB, moesin and STIM-1, showing overall an anti-oncogenic regulation of these 3 proteins following PAD-inhibitor treatment. In summary, our findings indicate PAD isozyme-specific regulation in the pro-oncogenic communication in GBM and highlight the potential of using PAD-isozyme specific inhibition for tailored treatment of GBM subtypes.

## 2. Results

### 2.1. PAD Isozyme-Specific Inhibitors Differently Modulate EV Release in LN18 and LN229 GBM Cells Following 1 h Treatment

**Figure 1:**
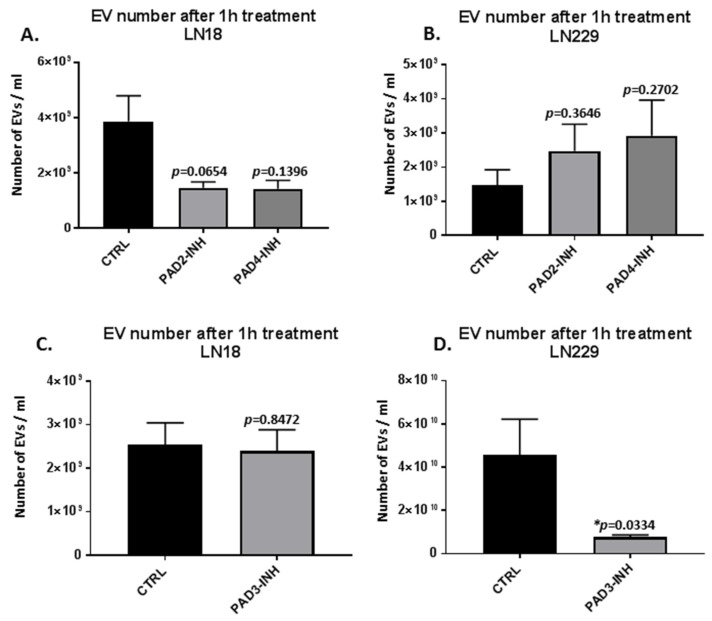
Peptidylarginine deiminase (PAD)2, PAD3 and PAD4 isozyme-specific inhibitor treatment shows glioblastoma multiforme (GBM) cancer cell line specific regulation of extracellular vesicle (EV) release. (**A**) Effects of PAD2 and PAD4 inhibitors on EV release in LN18 cells. (**B**) Effects of PAD2 and PAD4 inhibitors on EV release in LN229 cells. (**C**) Effects of PAD3 inhibitor on EV release in LN18 cells. (**D**) Effects of PAD3 inhibitor on EV release in LN229. (**D**). For each set of histograms, respectively, the PAD isozyme-specific inhibitor-treated and control-treated cells were run under the same experimental conditions. Exact *p*-values are indicated (* indicates significant differences with *p* < 0.05; *n* = 3 biological replicates for all).

Both LN18 and LN229 cells showed significant changes in EV release following 1 h PAD isozyme-specific inhibitor treatment, and this varied between the two cell lines (Figure 1). Both PAD2 and PAD4 inhibitors showed stronger effects on reducing EV release in LN18 cells (50%), although not reaching statistical significance (Figure 1A). In the LN229 cells on the contrary, both PAD2 and PAD4 inhibitor treatment resulted in some increased EV release, although not statistically significant (Figure 1B). After 1 h treatment with the PAD3 inhibitor, a significant reduction in numbers of EVs released was observed in the LN229 cells only (5-fold, *p* = 0.0334), while no significant change was observed in the LN18 cells. 

**Figure 2:**
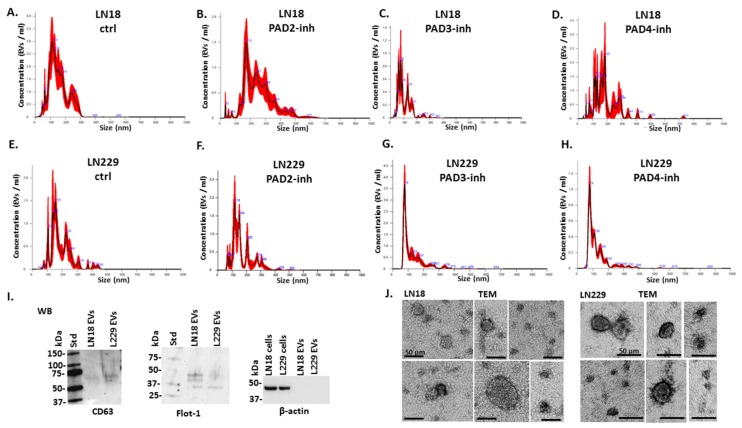
NTA size distribution profiles of EVs released from LN18 and LN229 cells following PAD isozyme-specific inhibitor treatment for 1 h and EV characterisation. Representative NTA profiles of LN18 cells following 1 h PAD inhibitor treatment (**A**–**D**): (**A**) Control DMSO treated cells; (**B**) PAD2 inhibitor treated cells; (**C**) PAD3 inhibitor treated cells; (**D**) PAD4 inhibitor treated cells. Representative NTA profiles of LN229 cells following 1 h PAD inhibitor treatment (**E**–**H**): (**E**) control DMSO treated cells; (**F**) PAD2 inhibitor treated cells; (**G**) PAD3 inhibitor treated cells; (**H**) PAD4 inhibitor treated cells. (**I**) Western blotting analysis (WB) showing that EVs isolated from LN18 and LN229 cells are positive for the EV specific markers CD63 and Flot-1; β-actin is absent from the EVs but present in the cells. (**J**) Transmission electron microscopy (TEM) images showing characteristic EV morphology for EVs isolated from both cell lines; the scale bar indicates 50 µm. In the NTA curves the black line represents the mean of the 5 repetitive readings per individual sample and the red line represents standard error (+/−) between those same 5 readings per sample. Each treatment group was measured in 3 biological replicates.

Figure 2 furthermore shows representative nanoparticle tracking analysis (NTA) profiles for EV size distribution of LN18 and LN229 control and PAD isozyme-specific treated GBM cells (Figure 2A–H), alongside characterisation of EVs by western blotting using the EV-specific markers CD63 and Flot-1; the absence of β-actin in EVs was assessed to rule out cell-contamination (Figure 2I). Typical morphology of EVs was verified by TEM (Figure 2J).

**Figure 3:**
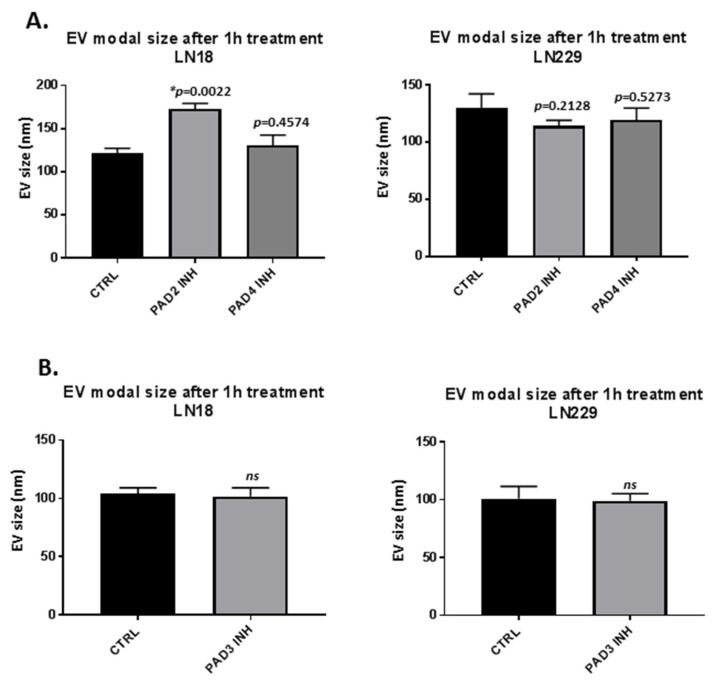
Effects of PAD2, PAD3 and PAD4 isozyme-specific inhibitor treatment on EV modal size in GBM cells, following 1 h treatment. (**A**) Modal size of EVs released from LN18 cells and LN229 cells, respectively, following 1 h PAD2 and PAD4 inhibitor treatment. (**B**) Modal size of EVs released from LN18 cells and LN229 cells, respectively, following 1 h PAD3 inhibitor treatment. Exact *p*-values are indicated, error bars show SD (* indicates significant differences with *p* < 0.05; ns = non-significant change; *n* = 3 biological replicates for all).

EV modal size was overall not affected by any of the PAD inhibitors following 1 h treatment (Figure 3A,B), except for some increase observed in EV modal size (from 125 nm to 175 nm) following 1 h treatment with the PAD2 inhibitor in LN18 cells (*p* = 0.0022) (Figure 3A).

### 2.2. MicroRNA EV-cargo is Differently Modulated in Response to 1 h PAD Isozyme-Specific Inhibitor Treatment in LN18 and LN229 GBM Cells

**Figure 4:**
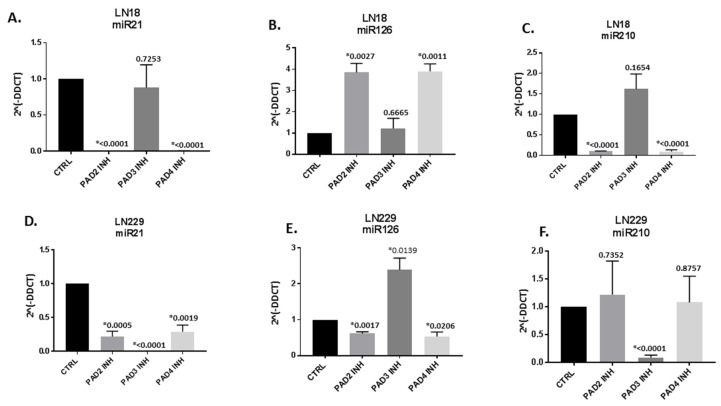
PAD isozyme-specific inhibitor mediated effects on EV microRNA cargo is GBM cell line specific. LN18 cell-derived EVs following 1 h PAD inhibitor treatment are shown in A–C: (**A**) PAD2, 3, and 4 isozyme-specific inhibitor-mediated effects on the pro-oncogenic miR21 in EVs derived from LN18 cells. (**B**) PAD2, 3, and 4 isozyme-specific inhibitor mediated effects on the anti-oncogenic miR126 in EVs derived from LN18 cells. (**C**) PAD2, 3, and 4 isozyme-specific inhibitor mediated effects on the hypoxia-related and pro-oncogenic miR210 in LN18 cells. LN229 cell-derived EVs following 1 h PAD inhibitor treatment are shown in D–F: (**D**) PAD2, 3, and 4 isozyme-specific inhibitor-mediated effects on the pro-oncogenic miR21 in EVs derived from LN229 cells. (**E**) PAD2, 3, and 4 isozyme-specific inhibitor mediated effects on the anti-oncogenic miR126 in EVs derived from LN229 cells. (**F**) PAD2, 3, and 4 isozyme-specific inhibitor mediated effects on the hypoxia-related and pro-oncogenic miR210 in LN229 cells. Results are represented as relative miR expression compared to the internal control miRs (2^Λ^(−DDCT)) and normalised to expression in control-treated cells; exact *p*-values are indicated, error bars show SD (*indicates significant differences with *p* < 0.05; *n* = 3 biological and 3 technical replicates for all).

When assessing EV cargo for pro-cancerous, GBM and hypoxia related microRNAs (miR21, miR126, miR210), respectively, some significant expression changes were observed, specific to the two cell lines and in response to the different PAD inhibitors (Figure 4). In LN18 cells, PAD3 inhibitor had no significant effects while both PAD2 and PAD4 inhibitors significantly changed EV miR cargo as follows: pro-cancerous miR21 was significantly reduced by PAD2 and PAD4 inhibitors in LN18 cells by 1055-fold and 131-fold, respectively (Figure 4A); the GBM protective microRNA marker miR126 was significantly increased by 3.8-fold and 3.9–fold following PAD2 and PAD4 inhibitor treatment, respectively (Figure 4B); and the hypoxia related miR210 was significantly reduced by 9.8-fold and 10.6-fold in LN18 cell-derived EVs following PAD2 and PAD4 inhibitor treatment, respectively (Figure 4C). Overall, PAD3 inhibitor was more effective in the LN229 cells and significantly reduced miR21 by 535-fold (Figure 4D); significantly increased miR126 by 2.4-fold (Figure 4E); and significantly reduced miR210 by 11.4-fold in LN229 cell-derived EVs (Figure 4F). In LN229 cells, both PAD2 and PAD4 inhibitor also had some significant anti-oncogenic effect by reducing miR21 by 4.6 and 3.4-fold, respectively (Figure 4D), but did neither up-regulate miR126 (protective in GBM) nor have significant effects on miR210 in cell-derived EVs (Figure 4F).

### 2.3. PAD Isozyme-Specific Inhibitors Affect PHB, STIM-1 and Moesin Protein Expression Differently in LN18 and LN229 GBM Cells Following 1 h Treatment

**Figure 5:**
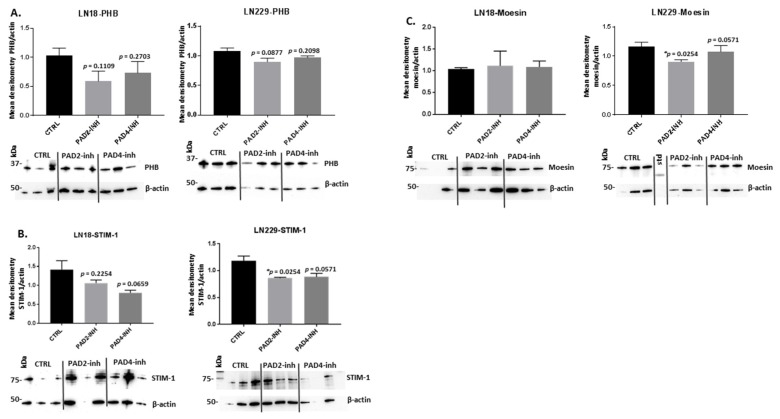
PAD2 and PAD4 isozyme-specific inhibitor 1 h treatment affects prohibitin (PHB), stromal interaction molecule 1 (STIM-1) and moesin protein levels in LN18 and LN229 GBM cell lines. (**A**) PHB protein levels in LN18 and LN229 cells, respectively, following 1 h treatment with the PAD2 and PAD4 inhibitors, compared to control-treated cells. (**B**) STIM-1 protein levels in LN18 and LN229 cells respectively, following 1 h treatment with the PAD2 and PAD4 inhibitors, compared to control-treated cells. (**C**) Moesin protein levels in LN18 and LN229 cells, respectively, following 1 h treatment with the PAD2 and PAD4 inhibitors, compared to control-treated cells. Representative blots are shown; the density ratios of the various proteins analysed and actin are presented as normalised quantified data (mean ±S.D.) for treatment with the PAD2 and PAD4 specific inhibitors, compared to DMSO treated controls. The corresponding molecular weight size standard is indicated in kilodaltons (kDa) on each blot. Exact *p*-values are indicated, error bars show SD (* indicates significant differences with *p* < 0.05; *n* = 3 biological replicates for all).

Following 1 h treatment with PAD2, PAD3, and PAD4 isozyme-specific inhibitors, respectively, the protein levels of PHB, STIM-1 and moesin were assessed by western blotting (Figure 5). The levels of PHB were somewhat reduced in LN18 cells both by PAD2 inhibitor (7% to 66%) and PAD4 inhibitor (1% to 49%), although not reaching statistical significance (Figure 5A); a similar pattern was seen in LN229 cells, with some reduction for PAD2 (7% to 21%) or PAD4 (19% to 80%) inhibitor (Figure 5A), but not reaching statistical significance. For effects on STIM-1 protein levels, both cell lines showed some reduction in this invasion protein following PAD2 and PAD4 inhibitor treatment. In LN18 cells, a 15% to 90% reduction (albeit non-significant) in STIM-1 was observed following PAD2 inhibition, while following PAD4 inhibition a significant (*p* = 0.0374) 33% to 90% reduction of STIM-1 protein levels was observed (Figure 5B). For LN229 cells, a significant (*p* = 0.0254) 29% to 34% reduction was seen in STIM-1 protein levels following PAD2 inhibition, while PAD4 inhibitor treatment resulted in 17% to 39% reduction in STIM-1 protein levels, almost reaching significance (*p* = 0.0571). For changes in moesin protein levels, neither PAD2 nor PAD4 inhibitor resulted in significant changes in moesin protein levels in LN18 cells, while in LN229 cells, moesin protein levels were significantly (*p* = 0.0163) reduced by 39% to 49% following PAD2 inhibitor treatment and by 13% to 27% (but non-significant) following PAD4 inhibitor treatment (Figure 5C).

**Figure 6:**
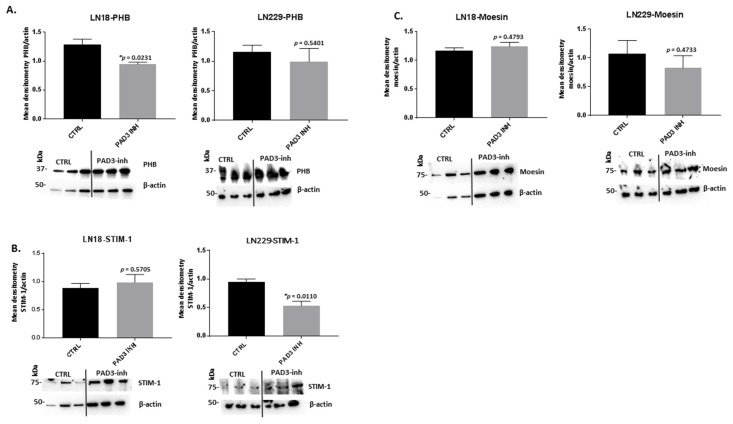
PAD3 isozyme-specific inhibitor 1 h treatment affects PHB, STIM-1 and moesin protein levels in two GBM cell lines. (**A**) PHB protein levels in LN18 and LN229 cells, respectively, following 1 h treatment with the PAD3 inhibitor, compared to control-treated cells. (**B**) STIM-1 protein levels in LN18 and LN229 cells, respectively, following 1 h treatment with the PAD3 inhibitor, compared to control-treated cells. (**C**) Moesin protein levels in LN18 and LN229 cells, respectively, following 1 h treatment with the PAD3 inhibitor, compared to control-treated cells. Representative blots are shown; the density ratios of the various proteins analysed and actin are presented as normalised quantified data (mean ±S.D.) for treatment with the PAD3 specific inhibitor, compared to DMSO treated controls. The corresponding molecular weight size standard is indicated in kilodaltons (kDa) on each blot. Exact *p*-values are indicated, error bars show SD (* indicates significant differences with *p* < 0.05; *n* = 3 biological replicates for all).

Following PAD3 inhibitor treatment, PHB protein levels were significantly reduced in LN18 cells by 27% to 38% (*p* = 0.0231), and up to 45% in LN229 cells, albeit non-significantly, following 1 h treatment (Figure 6A). PAD3 inhibitor did not significantly change STIM-1 protein levels in LN18 cells but resulted in significantly reduced STIM-1 protein levels in LN229 cells (26% to 53%; *p* = 0.0110) (Figure 6B). Moesin protein levels were not significantly affected by PAD3 inhibitor either in LN18 or LN229 cells, although a 1% to 68% (but non-significant) reduction was observed in LN229 cells (Figure 6C). 

Besides assessing a ranging effect of the three isozyme-specific inhibitors on changes of total protein levels of PHB, moesin and STIM-1, all three proteins were verified to be post-translationally deiminated as assessed by blotting the F95 protein eluates from both cell lines with the PHB, moesin and STIM-1 antibodies (Supplementary Figure S1B). Furthermore, FoldIndex© analysis revealed a number of disordered regions in moesin and STIM-1 (Supplementary Table S1).

### 2.4. PAD Isozyme-Specific Inhibitors Differently Affect Invasion in LN18 and LN229 GBM Cells

**Figure 7:**
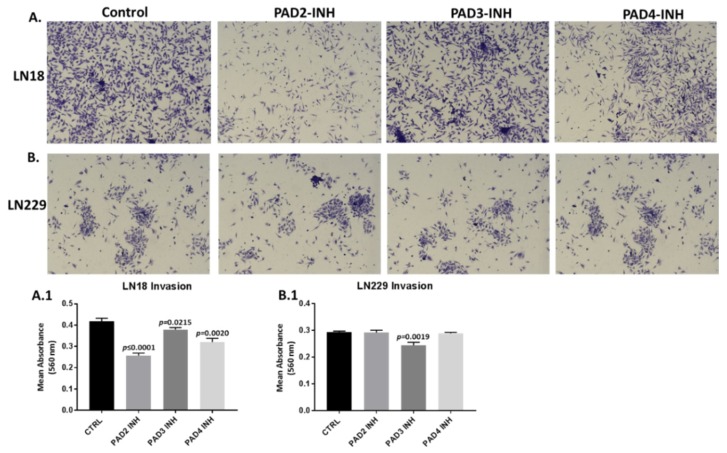
Cell invasion is differently affected by PAD isozyme-specific inhibitors in LN18 and LN229 GBM cells. Representative images of LN18 (**A**) and LN229 (**B**) cells following 16 h treatment with the three PAD-isozyme specific inhibitors, compared to control (DMSO)-treated cells following crystal violet treatment (imaged using a 10× objective). **A.1.** The corresponding histogram for the MTT assay for LN18 cell invasion following 16 h treatment with all three PAD isozyme-specific inhibitors. **B.1.** The corresponding histogram for the MTT assay for LN229 cell invasion following 16 h treatment with all three PAD isozyme-specific inhibitors. Exact *p*-values are indicated, and error bars show SD (*n* = 3 biological and 3 technical replicates for all).

**Figure 8:**
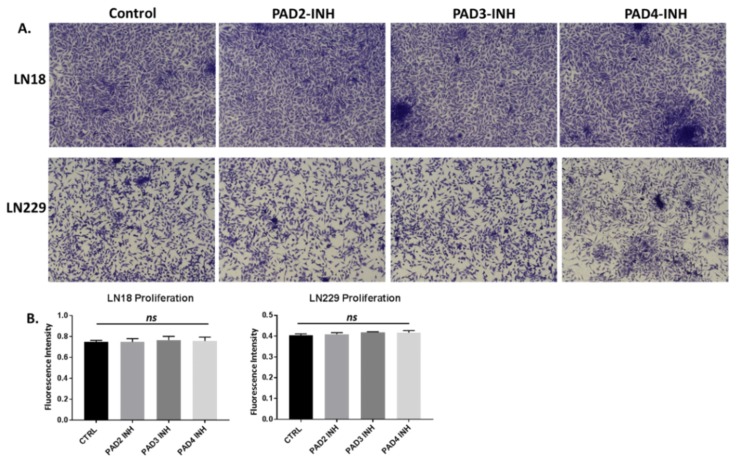
Cell proliferation assay for PAD isozyme-specific inhibitors in LN18 and LN229 GBM cells. (**A**). Representative images of LN18 and LN229 cells following 16 h treatment with the three PAD isozyme-specific inhibitors, compared to control (DMSO)-treated cells. (**B**) (imaged using a 10× objective). Chresyl violet assay revealed no significant effects (ns) of any of the three inhibitors on cell proliferation, following 16 h incubation, compared to control (DMSO)-treated cells (*n* = 3 biological and 3 technical replicates for all).

The invasiveness of LN18 and LN229 cells was studied in Boyden chambers with Matrigel. LN18 cells demonstrated noticeable invasion over 16 h (Figure 7A, A.1), while invasiveness of LN229 cells was far lower compared to that observed for LN18 cells. Incubation for 16 h with PAD2 and PAD4 inhibitors resulted in a significant suppression of invasiveness in LN18 cells by 39.3% (*p* ≤ 0.0001) and 23.2% (*p* = 0.0020) respectively, while less effect was observed following treatment with the PAD3 inhibitor (9.1%, *p* = 0.0215) (Figure 7A, A.1; *n* = 3). Cellular invasion of LN229 cells was overall lower than for LN18 cells (Figure 7B, control panel), and was significantly suppressed only by the PAD3 inhibitor (16.5%, *p* = 0.0019) (Figure 7B, B.1; *n* = 3), while neither PAD2 nor PAD4 inhibitors showed any significant effect on invasion in LN229 cells (0.4% and 1.25%, respectively; ns; *n* = 3) (Figure 7B, B.1). There was no significant change in cell proliferation over the 16 h incubation time with the PAD inhibitors for either cell line, compared to control treated cells (Figure 8A,B; *n* = 3). 

### 2.5. Deiminated Protein Targets and KEGG Networks Enriched in Deiminated Proteins Differ in LN18 and LN229 GBM Cells under Standard Culture Conditions

**Figure 9:**
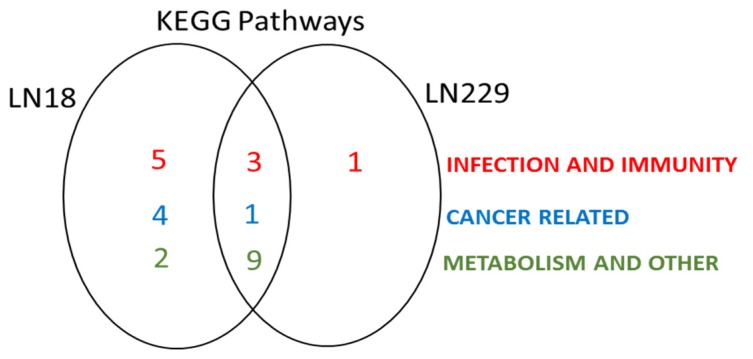
Deiminated Kyoto Encyclopedia of Genes and Genomes (KEGG) protein networks differ between LN18 and LN229 GBM cells. A Venn-diagram representing shared and different KEGG pathways identified to be enriched in deiminated proteins in the two GBM cell lines under study (LN18 and LN229), under standard culture conditions. For detailed network analysis highlighting the different KEGG pathways in both cell lines see Supplementary Figures S3 and S4.

STRING analysis revealed some common deiminated KEGG pathways (glycolysis/gluconeogenesis, ribosome, splicosome, protein processing in ER, carbon metabolism, oestrogen signalling pathway, biosynthesis of amino acids, arrhythmogenic right ventricular cardiomyopathy, antigen processing and presentation, Huntington’s disease, pathogenic *Escherichia. coli* (*E. coli*) infection) in both GBM cell lines, while several pathways were enriched for deiminated proteins in LN18 cells only (gap junction, aminoacyl-tRNA biosynthesis, pentose phosphate pathway; phagosome, necroptosis, Epstein–Barr virus infection, legionellosis, salmonella infection, mRNA surveillance pathway, longevity regulating pathway, central carbon metabolism in cancer, HIF-1 signalling pathway, thyroid hormone synthesis) or in LN229 cells only (pyruvate metabolism, IL-17 signalling pathway). Overall, more protein hits were identified as deiminated in LN18 cells (417 protein hits; Supplementary Table S2) compared to in LN229 cells (300 hits; Supplementary Table S3). KEGG pathways for deiminated proteins identified in both cell lines are presented in Supplementary Figures S3 and S4 for LN18 and LN229 cells, respectively (see also Supplementary Tables S2 and S3 for full LC-MS/MS data analysis of all protein hits). The Venn diagram in Figure 9 summarises common and distinct KEGG pathways enriched in deiminated proteins in LN18 and LN229 cells under normal culture conditions (see Supplementary Figures S3 and S4 for protein–protein interaction networks showing all identified KEGG pathways enriched in deiminated proteins).

## 3. Discussion

Regulation of EV release is critical for cellular communication and modulation of the microenvironment in cancer progression and metastasis and recognised to be of increasing importance in GBM [21,22]. GBM-derived EVs have furthermore been shown to induce tumour-promoting transformation of subventricular zone resident neural stem cells, possibly contributing to GBM recurrence [64]. Differences in EV release profiles between GBM cell lines, including EV sub-populations and during normal growth conditions, have previously been reported by us and others [9,38,65]. Several studies have assessed various types of EV cargo in GBM, including protein and genetic material [9,20,38,66,67,68]. Due to a range of pro-oncogenic cargo components transported by GBM cells for intra-tumour communication and the penumbra, including evidence for increased pro-oncogenic EV-release in response to standard chemotherapeutic treatment with TMZ [24], adjunct therapies that can modulate EV release and result in anti-oncogenic signatures, irrespective of or in conjunction with changes in EV numbers released, are of considerable interest. In the current study, both significant reduction in EV release, as well as anti-oncogenic modulation of microRNA expression following PAD isozyme-specific inhibitor treatment was observed in the two GBM cell lines, representative of a chemo-resistant (LN18) and chemo-sensitive (LN229) type [69]. A slight but significant increase in EV modal size was furthermore observed in LN18 cells following PAD2 inhibitor treatment and may have some relevance in cellular communication which will need further exploration, although aggregation of EVs contributing to this shift in modal size identified by NTA analysis cannot be excluded at this stage. Effects of the PAD isozyme-specific inhibitors on the modulation of microRNA EV-cargo highlight approaches for targeted modulation of EV cargo to change GBM intra- and inter-tumour communication. 

Both microRNAs and short non-coding RNAs have been identified in GBM EVs, with miR21 being one of the highest expressed microRNAs [20]. Changes in miR21 have been shown to affect viability, senescence and invasion in GBM [70,71,72], with miR21 silencing leading to decreased tumour size and improved survival in GBM animal models [73]. Furthermore, exosomes engineered to suppress miR21 were recently shown to reduce tumour volume in vivo in a rat GBM model [74]. Inhibition of miR21 has also been shown to enhance chemo-sensitivity of TMZ-resistant GBM cells in vitro [75]. We recently identified that miR21 was significantly reduced in LN18 and LN229 cells following pan-PAD inhibitor treatment and identified in the current study that the PAD2 and PAD4 inhibitors were most potent at reducing relative expression of miR21 in EV cargo of LN18 cells, while PAD3 inhibitor showed strongest relative reduction in miR21 expression in LN229 cell-derived EVs, out of the three PAD inhibitors tested. This also correlated with that PAD2 and PAD4 inhibitor most significantly reduced cell invasion in LN18 cells, as assessed by Matrigel™ Invasion Chamber assay, while only PAD3 inhibitor significantly affected cell invasion in LN229 cells. 

In GBM-derived patient samples, miR126 has been found to be decreased and related to high histopathological grades; while higher intra-tumoural miR126 levels are indicative of significantly improved survival duration, compared to patients with lower miR126 levels [76]. Over-expression of miR126 has been shown to suppress glioma cell proliferation and invasion in vitro [77]. Previously we found that miR126 was significantly elevated in GBM cells and cell-derived EVs following pan-PAD inhibitor treatment and have in the current study identified that in LN18 cell-derived EVs PAD2 and PAD4 inhibitors were significantly effective in raising relative miR126 expression, while PAD3 specific inhibitor was not effective in increasing relative miR126 expression in LN18 cell-derived EVs. In LN229 cell-derived EVs, only PAD3 inhibitor significantly increased relative levels of miR126 while PAD2 and PAD4 inhibitors slightly (but significantly) decreased miR126; therefore, only PAD3 inhibitor achieved the “protective” anti-oncogenic response by elevating relative levels of miR126 in LN229 cell-derived EVs. Interestingly, cannabidiol, which is effective in GBM treatment [78,79] and was also recently identified as an potent EV modulatory agent [37,38], has also been found to elevate miR126 and reduce miR21 EV-cargos from LN18 and LN229 cells, possibly explaining some of its protective functions in GBM [37].

miR210 has previously been identified as a major miR induced under hypoxia and has important roles in mitochondrial metabolism, DNA damage response, cell proliferation and apoptosis [80]. MiR210 is furthermore implicated in the regulation of cell glycolytic activity, is linked to inflammation [81] and is also involved in angiogenesis and vascular remodelling [82], including via EV-mediated transport [83]. miR210 has been identified as a regulator of the hypoxia pathway [84,85] and belongs to a group of hypoxia-mediated microRNAs, which are upregulated in glioma compared to normal brain tissue [86] and upregulated in patient-derived glioblastoma spheroids upon hypoxia exposure [87]. Elevated plasma expression of miR210 has been found in GBM compared to low-grade astrocytoma patients [88], as well as being elevated in serum and associated with tumour grade and poor patient outcome [62]. miR210 is also elevated and associated with worse prognosis in higher grade GBM tumours, compared to lower grade gliomas [61] and promotes hypoxic survival and chemoresistance in GBM cells [89]. Furthermore, miR210 has recently been found to belong to miRs that encourage radioresistance of GBM [63]. In the current study PAD2 and PAD4 inhibitors significantly reduced relative miR210 levels in LN18 cell-derived EVs, while in LN229 cell-derived EVs only the PAD3 inhibitor significantly reduced relative miR210 levels. 

The observed decrease in pro-oncogenic miR21, hypoxia related and pro-oncogenic miR210 and the increase in anti-oncogenic miR126 levels in GBM cell-derived EVs, caused by the different PAD isozyme-specific inhibitors in the current study, indicates GBM cell type selective anti-GBM functions of the different PAD isozyme-specific inhibitors. These observations highlight heterogeneity of PAD isozyme expression in different GBM cell lines.

Modulation of proteins involved in cancer invasion and progression is of pivotal importance for targeting cancer, including GBM. Targeted modulation of the invading GBM tumour edge, which can act as a seed for recurrence [90], is therefore of considerable importance. New strategies for regulation of invasion-proteins including via inhibition of post-translational deimination, which may facilitate, for example, actin assembly and proteins involved in filopodia formation, as well as targeting proteins linked to stem-ness in the tumour periphery, promoting tumour invasion and rapid tumour progression, may therefore be of interest. 

Previously we established that pan-PAD inhibitor Cl-amidine modulated cancer promoting proteins, including in GBM [9,33]. Prohibitin is a multifaceted protein involved in cell survival and apoptosis and a key mitochondrial house-keeping protein [41,44]. We had previously identified that pan-PAD inhibitor Cl-amidine reduced PHB expression somewhat following 1 h treatment, in addition to affecting post-translational deimination, in the two GBM cell lines under study [9], although such effects were more pronounced in the LN18 cell line [9]. In the current study, PAD isozyme-specific inhibition highlighted differences between the two GBM cell lines and PAD isozyme-specificity on regulation of PHB protein levels, following 1 h treatment. In LN18 cells, PAD3 inhibitor led to a significant reduction in PHB levels, correlating with previous findings that Cl-amidine (pan-PAD inhibitor with most specificity for PAD3) affected PHB levels in this GBM cell line [9]. Both PAD2 and PAD4 inhibitor less effectively modified PHB levels in LN18 cells, with PAD2 inhibitor somewhat more effective in lowering PHB levels (albeit not significantly) than PAD4 inhibitor, following 1 h treatment. In LN229 cells PHB reduction was more pronounced following 1 h treatment with PAD2 and PAD3 inhibitors, although not significant and overall less than for LN18 cells. This correlates with previous findings showing non-significant changes in PHB levels in LN229 cells using pan-PAD inhibitor Cl-amidine for the same time duration [9]. 

STIM-1 was previously identified to be a deiminated protein candidate in GBM cells by our group [9], and folding dynamics of STIM-1 have been discussed with respect to adaption to differential roles in Ca^2+^ homeostasis and signalling [45]. Roles for STIM-1 in Ca^2+^ entry mechanisms have been highlighted in a range of pathophysiological processes including infection and cancer [91]. As STIM-1 plays roles in Ca^2+^ regulation, it also has effects on EV release, which is in part a Ca^2+^ mediated mechanism and furthermore may impact mitochondrial function. Reduction in STIM-1 protein expression observed in the current study, following PAD isozyme-specific inhibitor treatment, correlates with a significant reduction observed in EV release. STIM-1 activity has indeed been shown to be essential for GBM invasion [46]. In studies on GBM human U251 and rat C6 cells, STIM-1 knockdown has been shown to reduce Ca^2+^ influx and to inhibit tumour cell proliferation and induce apoptosis [92]. Higher STIM-1 expression is correlated with migration, tumour size and clinical outcome in cervical cancer [93] and such correlation with tumour size is also seen in breast cancer [94]. Furthermore, STIM-1 enhances cell migration and promotes metastasis of cancer cells [93,95]. The findings reported in the current study, showing reduction of STIM-1 protein following PAD isozyme-specific inhibitor treatment, align with these previous studies as the PAD inhibitors lead to reduced STIM-1 protein and also to reduced cell migration, as assessed by reduced invasion observed for GBM cells following PAD inhibitor treatment. In LN18 cells, PAD4 inhibitor was most effective at lowering STIM-1 levels, while in LN229 cells PAD3 inhibitor was significantly and most effective, with PAD2 and PAD4 inhibitor leading to some (but not significant) reduction of STIM-1. 

Moesin is an ezrin-radixin-moesin (ERM) family member and involved in the regulation of cell adhesion, polarity and migration [96]. Moesin connects the actin cytoskeleton to transmembrane receptors, and its upregulation is correlated with increased cell invasion and migration in GBM [49]. Moesin has been associated with filopodia formation, which are dynamic actin-rich membrane protrusions important for cell adhesion, membrane trafficking (including EV internalisation) [48] and therefore also of importance in cancer cell adhesion and invasion [97,98,99]. The correlation of moesin overexpression with higher grade GBM has also been related to its ability to increase stem cell neurosphere formation [50,100], which may furthermore promote stem-ness (which correlates with aggressiveness and chemo-resistance) in GBM. Increased moesin expression is related to invasion and metastasis and is also correlated to a progressive pathological state of pancreatic [101] and breast cancer [102]. In the current study PAD isozyme-specific inhibition resulted in some reduced moesin levels, which differed between the two GBM cell lines and PAD isozyme-specific inhibitor treatment. Moesin was not reduced by any PAD-isozyme inhibitor treatment in the LN18 cells, which overall have been reported to also be more chemo-resistant [69], while in LN229 cells PAD2 and PAD3 inhibitor lowered moesin levels following 1 h treatment. 

It must be noted that the current study assessed changes in total protein levels only, following 1 h PAD inhibitor treatment of the three proteins under study, while PAD inhibition may also affect changes in post-translational deimination of the proteins, affecting protein–protein interactions, and this will need further detailed evaluation, including at longer time-points. The results of the invasion cell assay were assessed after a longer time-point, following 16 h, and do correlate with the anti-oncogenic signatures observed following 1 h PAD isozyme-specific treatment. Overall, more target proteins were found to be deiminated in LN18 cells compared to LN229 cells, and this also correlated with more significant effects of PAD inhibition on invasion in LN18 cells. We verified that all three proteins under study are post-translationally deiminated in both cell lines (Supplementary Figure S1B) while FoldIndex© analysis [103] furthermore shows that both STIM-1 and moesin are highly unfolded proteins (Supplementary Table S1), which makes them susceptible for post-translational deimination [40,104,105]. While PHB does not show any unfolded regions, a high number of arginines are present, and these pose as sites for citrullination/deimination (Supplementary Table S1). Furthermore, we previously showed that PHB deimination was reduced in GBM cells following 1 h pan-PAD inhibition [9]. Further in-depth investigation into roles for the most critical arg/cit conversions in the context of PAD-inhibitor treatment and PAD isozyme-specific preferences for deimination of target proteins could for example be explored using site-directed mutagenesis in selected proteins. 

Our findings indicate differences in function of PAD isozymes 2, 3 and 4 in the two GBM cell lines, also differently affecting pro-oncogenic protein expression. PAD isozyme-specific inhibitor treatment in the two GBM cell lines reflects to some extent the finding that PAD2 and PAD4 are higher expressed in LN18 cells compared to PAD3, while PAD3 is higher expressed in LN229 cells compared to PAD2 and PAD4 [9] (Supplementary Figure S1A). This is in accordance with the PAD3 inhibitor being most effective at reducing EV release from LN229 cells and most effectively reducing STIM-1 protein levels in LN229 cells. Furthermore, PAD3 inhibitor most effectively modulated the three microRNAs under study to an anti-oncogenic signature in the LN229 cell-derived EVs. A similar trend was observed for LN18 cells, where PAD2 and PAD4 inhibitors most effectively reduced EV release (while PAD3 inhibitor had no significant effect) and also significantly modulated the three microRNAs to an anti-oncogenic signature in the cell-derived EVs. The PAD-inhibitor mediated effects on reducing protein levels of STIM-1, moesin and PHB, all pro-oncogenic proteins, was less prominently pronounced, although overall a trend for PAD2 and PAD4 inhibitors was observed in LN18 and for the PAD3 inhibitor in LN229 cells. The PAD isozyme-specific inhibitors affected cell invasiveness differently in the two GBM cell lines and were overall more effective in reducing invasiveness in LN18 cells, with PAD2 inhibitor being most effective but overall all three PAD inhibitors showing significantly reduced invasion of LN18 cells, while in LN229 cells only PAD3 inhibitor significantly reduced invasion following 16 h treatment. Furthermore, relative expression of miR21, involved in cell invasion, was most significantly reduced by PAD2 and PAD4 inhibitors in EV cargo from LN18 cells and by the PAD3 inhibitor in EV cargo from LN229 cells. Overall, this indicates isozyme-specific selection of target proteins and miRs involved in invasion, also as PAD isozyme expression significantly differed between the two cell lines (Supplementary Figure S1A; [9]). In both cell lines, the PAD inhibitors seemed to promote invasiveness independently of proliferation. This furthermore highlights current challenges in the treatment of GBM, which are known to be a highly heterogeneous group of tumours. 

In both GBM cell lines under study, enrichment of deiminated proteins in a number of KEGG pathways was identified under normal culture conditions. Some pathways were common to both cell lines (including glycolysis/gluconeogenesis, ribosome, splicosome, oestrogen signalling pathway, carbon metabolism, bacterial infection, antigen processing and presentation, Huntington’s disease), while some KEGG pathways differed between the two cell lines. Interestingly, deiminated KEGG pathways for HIF-1 were identified in LN18 cells only, but not in LN229 cells, under normal conditions, and this may be indicative of some differences in hypoxia regulation via post-translational deimination. HIF-1 expression plays a major role in GBM development and progression, participating in glucose uptake, cancer proliferation, cell mobility, cancer stem cell metabolic reprogramming and chemo-resistance [106,107,108,109]. Inhibition of HIF-1 has been shown to induce cell death of GBM [110], and HIF-1 over-expression and silencing studies have furthermore shown regulation of specific miRNAs, including miR210, to be HIF-1 dependent [89]. To what extent deimination of these pathways plays roles in the regulation of hypoxia resistance and chemo-resistance remains to be further investigated. Interestingly, deimination of HIF-1 related KEGG pathways has recently been described in animal models of cancer resistance and hypoxia tolerance [56,57]. Furthermore, KEGG pathways for thyroid hormone synthesis were identified to be enriched for deiminated proteins in LN18 cells only. Thyroid hormone synthesis has been suggested as one of the pathogenic factors of GBM, with implications in formation and course of GBM pathogenesis [111,112]. LN18 cells also showed enrichment for deiminated proteins in KEGG pathways for Epstein–Barr virus infection (EBV). The relationship between EBV and GBM still remains to be fully understood [113] and has been recognized to be associated with worse patient prognosis [114]. In LN18 cells, deiminated proteins were enriched for the necroptosis pathway, which has been highlighted as a novel therapeutic target for GBM, as necrosis seems to be related to GBM proliferation, angiogenesis and invasion [115]. As necroptosis is a pathological and radiological hallmark of GBM [116], it has been proposed that modulating necroptosis in GBM could circumvent apoptosis resistance, which is common in GBM [115,117,118]. Therefore, regulation via post-translational deimination of these pathways, which could be targeted via selective PAD-inhibitors, may be of some interest. KEGG pathways for phagosome were also found to be enriched in protein deimination in LN18 cells. Phagosome KEGG pathways have been identified to be associated with TMZ resistance in GBM at the proteomic level [119] and related to the progression of glioma into glioblastoma by gene expression profiling [120]. Therefore, regulation via deimination may also be of considerable interest, particularly as this pathway was detected in LN18 cells, which are reported to be the more chemo-resistant of the two GBM cell lines [69]. While KEGG pathways for glycolysis, which is of high relevance for GBM [121], were identified to be deiminated in both GBM cell lines, the pentose phosphate pathway (PPP) was identified as a deimination KEGG pathway in LN18 cells only and the KEGG pathway for pyruvate metabolism in LN229 only. Both are related to glycolysis, with the PPP associated with cell migration and glial tumour aggressiveness [122,123]. Pyruvate plays a key role in GBM metabolic reprogramming [124] and modulation of the pyruvate pathway induces alterations of metabolic and stress-related pathways in GBM [121] also with putative roles in hypoxia-dependent resistance of GBM [110]. While carbon metabolism KEGG pathway was identified for deiminated proteins in both GBM cell lines, KEGG pathway for central carbon metabolism in cancer was identified in LN18 cells only. Carbon metabolism is related to metabolic adaption and growth in GBM [125,126], including in TMZ resistance [127] and in the control of glioma cell transformation to a higher aggressive stage [109]. Roles for post-translational deimination in such metabolic control have not been investigated and may be of relevance as this pathway was prominent for deimination in LN18 cells, which present a more aggressive and chemo-resistant form than LN229 [69]. Interestingly, enrichment of deiminated proteins in carbon metabolism KEGG pathway has also been recently recognized in whales [57], which are hypoxia tolerant and long-lived mammals displaying cancer resistance [128]. Only LN229 cells showed enrichment of deiminated proteins in IL-17 KEGG pathway, and this was not found in LN18 cells. Regulation of IL-17 via post-translational deimination may be of interest for recent IL-17 inhibitor treatment approaches, which were shown to decrease GBM tumour hypoxia, angiogenesis and tumour growth in animal models [129]. Furthermore, KEGG pathways for bacterial infection were identified to be deiminated in both GBM cell lines (pathogenic *E. coli*) but more prominently for LN18 cells (*E. coli*, salmonella and legionellosis). This may be of relevance for success of using bacterial carriers for glioblastoma therapy [130] as well as for management of bacterial infection following GBM surgery and treatment [131,132,133,134]. Indeed, critical roles for PADs, including pan-PAD and PAD-isozyme specific regulation of bacterial membrane vesicle release, critical for host-pathogen interactions, and in bacterial drug resistance, have recently been described [135]. Furthermore, the modulation of the host’s immune proteins via post-translational deimination by bacterial arginine deiminases is also a recognized mechanism for immune evasion [136]. 

The differences identified in the current study in deiminated KEGG pathways between the two GBM cell lines may indicate selective differences in the preference of target proteins for deimination by the different PAD-isozymes, particularly as in LN18 cells PAD2 and PAD4 isozymes are more prominently expressed while PAD3 is found at higher levels compared to PAD2 and PAD4 in LN229 cells [9]. 

In summary, our findings indicate PAD isozyme-specific regulation in the pro-oncogenic communication in GBM and highlight the potential of using PAD-isozyme specific inhibition for tailored treatment in GBM subtypes.

## 4. Materials and Methods 

### 4.1. GBM Cell Cultures and PAD-Inhibitor Treatment

LN18 (ATCC^®^ CRL-2610™, grade IV glioblastoma derived from a male patient with a right temporal lobe glioma) and LN229 (ATCC^®^ CRL-2611™, glioblastoma derived from a female patient with right frontal parietal-occipital glioblastoma) were cultured according to ATCC’s recommendations, to 80% confluence in 75 cm^2^ flasks in complete Dulbecco’s Modified Eagle’s Medium (DMEM), with 10% foetal bovine serum (FBS) at 37 °C/5% CO_2_. Cells were split every 3–5 days, depending on confluence. The cell lines were chosen as an example of a chemo-resistant (LN18) and chemo-sensitive (LN229) GBM cell line respectively, according to previously published literature [69]. GBM cells were grown to 80% confluency before 1 h treatment with PAD2 (AMF30a, 5 μM; [137]), PAD3 (Cl-4 amidine, 10 μM; [138]) and PAD4 (GSK199, 10 μM; [139]) inhibitors, respectively, based on cell viability tests (see Section 4.2 and Supplementary Figure S2) and previously published literature [39,135,137,138,139]. The PAD inhibitors were dissolved in 0.001% DMSO), and DMSO (0.001%) treated cells were used as controls. For effects on EV release, protein and microRNA expression, cells were treated for 1 h with the PAD inhibitors, while for cell proliferation and invasion assays, treatment time was 16 h. For EV isolation, before application of the PAD inhibitors, the serum-containing medium was removed to avoid contamination of EVs from the FBS; the cells were washed in DPBS, and thereafter, serum-free medium containing the respective PAD-inhibitors (or corresponding DMSO control) were added for 1 h. Following 1 h incubation, the medium was removed from all treatments for EV isolation (Section 4.4), and the cells were trypsinised for subsequent protein extraction and western blotting (Section 4.7).

### 4.2. Cell Viability Assays following PAD Inhibitor Treatment 

Cell viability of LN18 and LN229 GBM cells was assessed after 1 h incubation with PAD2 (AMF30a, 5 μM), PAD3 (Cl4-amidine, 10 μM) and PAD4 (GSK199, 10 μM) inhibitors, respectively, compared to DMSO control-treated cells (Supplementary Figure S2). Glioblastoma cell lines LN18 and LN229 were seeded at a density of 1 × 10^4^ on to a 96 well plate (Nunc, Roskilde, Denmark) for 2–3 days. Cells were treated with either medium only, DMSO, PAD2 (AMF30a; 5 μM), PAD3 (Cl4-amidine; 5, 10, 50, 100 μM) and PAD4 (GSK199; 10 μM) inhibitors respectively, for 1 h at 37 °C, 5% CO_2_. PrestoBlue Cell Viability Reagent (ThermoFisher Scientific, Dartford U.K.) was added (1:10 dilution) to each well and incubated for 10 min at 37 °C, according to the manufacturer’s instructions (ThermoFisher). Fluorescence was measured using CLARIOstar plate reader (BMG Labtech, Aylesbury, Bucks, U.K.) at 545-20/600-40 nm.

### 4.3. Modulation of EV Release Using PAD2, PAD3 and PAD4 Isozyme-Specific Inhibitors Following 1 h Treatment

The effect of PAD2 (AMF30a; 5 μM), PAD3 (Cl4-amidine; 10 μM) and PAD4 (GSK199; 10 μM) specific inhibitors on EV release from GBM LN18 and LN229 cells was assessed following 1 h incubation time with the PAD2, 3 or 4 isozyme-specific inhibitors, respectively. LN18 and LN229 cells were cultured and maintaied in T75 flasks, in triplicates, in the presence of culture medium (pre-warmed DMEM, supplemented with 10% FBS; Sigma-Aldrich, Gillingham, U.K.), according to ATCC’s recommendations. LN18 and LN229 cells were grown to 80% confluency per T75 flask, whereafter the cells were split in culture medium (10 mL per T75 flask of pre-warmed DMEM, supplemented with 10% FBS; Sigma-Aldrich, U.K.) in preparation for each experiment, which then was carried out 2–3 days following splitting, and upon the cells in the flasks reaching 70% to 80% confluency. For EV isolation, treatment with the PAD isozyme-specific inhibitors and 0.001% DMSO, respectively, was carried out in biological triplicate per treatment as follows: Before PAD inhibitor (or DMSO control) treatment, the serum-containing medium was removed from the T75 flasks containing the cell preparations, to avoid contamination of EVs from the FBS in the medium, and the cells were washed three times with pre-warmed Dulbecco’s PBS (DPBS). Thereafter, fresh pre-warmed serum- and EV-free DMEM containing either the PAD inhibitors (dissolved in 0.001% DMSO, in 5 mL medium per T75 flask) or DMSO (0.001%, in 5 mL medium per T75 flask) were added. The cells were incubated for 1 h in the presence of the PAD-inhibitors (and DMSO control) at 37 °C/5% CO_2_. Following 1 h incubation time, the EV-containing media (5 mL per T75 flask) were collected from the flasks. Cell debris was removed by centrifugation at 200 g for 10 min, and thereafter, EVs were isolated from the remaining supernatant as described in Section 4.4. The PAD2-inhibitor and PAD4-inhibitor treatments with a corresponding DMSO control were run together; PAD3-inhibitor treatment and a corresponding DMSO control treatment were run together, as reflected in the histograms in Figure 1 and Figure 3.

### 4.4. EV Isolation and Quantification by Nanoparticle Tracking Analysis

EV isolation was carried out according to established protocols [9,36,38] and according to the recommendations of the International Society of Extracellular Vesicle Research (ISEV) [140]. Differential centrifugation was carried out on the cell culture supernatants (5 mL collected from each flask) as follows: First the supernatants were centrifuged at 4000 g for 30 min at 4 °C to remove cell debris, followed by centrifugation of the collected supernatant for 1 h/4 °C at 100,000 g. The supernatant was discarded and the isolated EV pellets were resuspended and washed in ice-cold DPBS, centrifuged again at 100,000 g for 1 h/4 °C, and thereafter, the final EV enriched pellet was resuspended in 100 μL sterile EV-free PBS. Nanoparticle tracking analysis (NTA) was carried out using the NS300 Nanosight (Malvern Panalytical Ltd., Malvern, U.K.), equipped with a sCMOS camera and a 405 nm diode laser, to enumerate the EVs. Samples were diluted 1:100 in sterile-filtered EV-free DPBS, and the number of particles in the field of view was maintained in the rage of 30–50 with a minimum concentration of samples at 5 × 10^7^ particles/mL. Camera settings were according to the manufacturer’s instructions (Malvern Panalytical Ltd.), recording five 60 s videos per sample and averaging the obtained replicate histograms. Each experiment was repeated in three biological replicates.

### 4.5. EV Characterisation by Transmission Electron Microscopy

Isolated EVs from LN18 and LN220 cells were resuspended in 100 mM sodium cacodylate buffer (pH 7.4). A drop (~3–5 μL) of the suspension was placed on to a grid with carbon support film, which had previously been glow discharged. When the suspension had partly dried, the grid was placed on a drop of solution of 2.5% glutaraldehyde in 100 mM sodium cacodylate buffer (pH 7.4) for 1 min and washed afterwards by touching it to the surface of three drops of distilled water. Excess water was removed by touching the grid to a filter paper. A small drop of stain (2% aqueous Uranyl Acetate; Sigma-Aldrich) was then applied to the grid. After 1 min, the excess stain was removed by touching the edge to a filter paper. The grid was dried at room temperature and thereafter the samples were viewed in TEM. Imaging was performed using a JEOL JEM 1400 transmission electron microscope (JEOL, Tokyo, Japan) operated at 80 kV at a magnification of 30,000 to 60,000. Digital images were recorded using an AMT XR60 CCD camera (Deben, Bury Saint Edmunds, U.K.).

### 4.6. Analysis of microRNAs miR21, miR126 and miR210 in GBM Cell EV-Cargo Following 1h PAD Inhibitor Treatment

For assessment of microRNA cargo in the GBM-derived EVs, LN18 and LN229 cells were cultured to 80% confluency in T75 flasks in DMEM supplemented with 10% FBS as before. The cells were washed with EV-free DPBS, and thereafter, fresh EV and serum-free medium was added, containing the PAD-isozyme specific inhibitors (same concentrations as before), and 0.001% DMSO for control treatment. After 1 h incubation time, the cell medium was collected for EV isolation. EVs were isolated as described above and thereafter processed for RNA isolation, cDNA translation and assessment for the relative expression of miR21, miR126 and miR210. RNA was extracted from treated and control-treated cells using Trizol (Sigma, U.K.), and RNA concentration and purity was measured using the NanoDrop Spectrophotometer (ThermoFisher Scientific, Dartford, U.K.) at 260 nm and 280 nm absorbance. RNA was reverse transcribed to cDNA using the qScript microRNA cDNA Synthesis Kit (Quantabio, Beverly, MA, USA) according to the manufacturer’s protocol. The resulting cDNA was used to assess the expression of microRNAs miR21, the main microRNA associated with pro-oncogenic function, miR126, associated with protective function in GBM, and miR210, associated with hypoxia and pro-oncogenic environment in GBM. U6-snRNA and hsa-let-7a-5p were used as a reference RNA for normalization of miR expression levels. The PerfeCTa SYBR^®^ Green SuperMix (Quantabio, USA) was used together with MystiCq microRNA qPCR primers for miR21 (hsa-miR-21-5p), mir126 (hsa-miR-126-5p) and miR210 (hsa-miR-210-5p), which were obtained from Sigma (U.K.). The sequences for U6-snRNA primers were U6 forward, 5′-GCTTCGGCAGCACATATACTAAAAT-3′ and hsa-let-7a-5p forward 5′-CCGAGCTGAGGTAGTAGGTTGTATA-3′ reverse 5′-CGCTTCACGAATTTGCGTGTCAT-3′ for both. The thermocycling conditions were as follows: denaturation at 95 °C/2 min, followed by 40 cycles at 95 °C/5 s and 60 °C/15 s and extension at 72° C/15 s. The miR21, miR126 and miR210 expression levels were normalized to that of U6 using the ΔΔCT method according to Livak and Schmittgen [141]. The experiments were carried out in 3 biological and 3 technical repeats.

### 4.7. Western Blotting Analysis

Total protein was extracted from treated and control-treated LN18 and LN229 cells, in the presence of RIPA+ buffer (Sigma, U.K.) containing 10% protease inhibitor complex (Sigma), pipetting gently with regular intervals while shaking the cell preparation on ice for 2 h. Thereafter, the cell preparations were centrifuged at 16,000 g (4 °C/20 min) and the supernatant containing the extracted proteins collected. The protein extracts were either used immediately for immunoprecipitation and proteomic analysis or re-constituted in 2 x Laemmli sample buffer for western blotting. Protein extracts from LN18 and LN229 cells, in 2 x Laemmli sample buffer containing 5% β-mercaptoethanol, were boiled for 5 min at 100 °C before separation by SDS-PAGE, using 4% to 20% Mini-Protean TGX protein gels (BioRad, Deeside, U.K.), followed by semi-dry western blotting analysis. Even transfer to nitrocellulose membranes (0.45 μm, BioRad) was assessed using Ponceau S staining (Sigma). The membranes were blocked for 1 h at room temperature (RT) in 5% BSA (Sigma) in Tris buffered saline (TBS) with 0.1% Tween20 (TBS-T), followed by overnight incubation at 4 °C with the following primary antibodies for the cell lysates (used 1/1000 in TBS-T): anti-PAD2 (ab50257, Abcam), anti-PAD3 (ab50246), anti-PAD4 (ab50332), anti-prohibitin (ab75771), anti-STIM-1 (ab57834), anti-moesin (ab52490). For characterization of EVs, isolated EVs were assessed by WB using the EV-specific markers CD63 (ab68418; 1/1000 in TBS-T) and Flot-1 (ab41927; 1/2000 in TBS-T). Following primary antibody incubation, membranes were washed in TBS-T, incubated for 1 h at RT with the corresponding HRP-conjugated secondary antibodies (anti-rabbit IgG, BioRad, U.K.), followed by TBS-T washes and visualisation using enhanced chemilumnicence (ECL; Amersham, U.K.) and the UVP BioDoc-ITTM System (Thermo Fischer Scientific, Hemel Hempstead, U.K.) HRP-conjugated anti-β-actin antibody (ab20272, Abcam, 1/5000 in TBS-T) was used as an internal loading control and for assessment for the purity of EV isolation (confirmed by absence of β-actin). Densitometry analysis of PHB, STIM-1 or moesin protein levels relative to the internal actin control was carried out, for assessment of changes in total protein levels between PAD-inhibitor treated and control-DMSO treated cells, using ImageJ [142].

### 4.8. Cancer Cell Invasion Assay

Cell invasion assay was performed as previously described in detail [143]. Briefly, 5 × 10^5^ cells (treated with the PAD isozyme-specific inhibitors or DMSO control as before) were plated on Matrigel-coated transwell filters (Corning™ BioCoat™ Matrigel™ Invasion Chamber with Corning™ Matrigel Matrix; BD Biosciences, Wokingham, Berkshire, U.K.) in a chemotactic gradient of 1:10% FBS. After 16 h incubation, the total number of invaded cells was determined by MTT assay (Abcam, Cambridge, U.K.) and further confirmed by crystal violet assay (Abcam, U.K.). In parallel, the same number of cells was plated and incubated for 16 h to determine the effect of the PAD isozyme-specific inhibitors on cell proliferation by MTT (3-(4,5-dimethylthiazol-2-yl)-2,5-diphenyl tetrazolium bromide) assay. Absorbance was measured using CLARIOstar plate reader (BMG Labtech, Aylesbury, U.K.) at 540–590 nm and normalised according to the control. The experiments were performed in 3 biological and 3 technical repeats.

### 4.9. Assessment of KEGG Pathways for Deiminated Proteins in LN18 and LN229 GBM Cells under Standard Culture Conditions

Protein extracts from LN18 and LN229, obtained by protein extraction using RIPA+ buffer, were added to mini-prep sepharose columns in the presence of the pan-deimination F95 antibody (MABN328, Merck, Nottingham, U.K.) for immunoprecipitation of total deiminated proteins using the Catch and Release^®^ v2.0 Immunoprecipitation Kit according to the manufacturer’s instructions (Merck, Nottingham, U.K.) F95 enrichment was carried out overnight at 4 °C on a rotating platform. and the F95 bound proteins were thereafter eluted using denaturing elution buffer (Merck), according to the manufacturer’s instructions. F95-enriched eluates from both GBM cell lines were thereafter analysed by liquid chromatography with tandem mass spectrometry (LC-MS/MS; Cambridge Proteomics, Cambridge, U.K.). For LC-MS/MS, the F95 enriched eluates were run 0.5 cm into a 15% TGX gel (BioRad, U.K.) and each cut out as one band. The 1D gel bands were transferred into a 96-well PCR plate. The bands were cut into 1 mm^2^ pieces, destained, reduced (DTT) and alkylated (iodoacetamide) and subjected to enzymatic digestion with trypsin overnight at 37 °C. After digestion, the supernatant was pipetted into a sample vial and loaded onto an autosampler for automated LC-MS/MS analysis. All LC-MS/MS experiments were performed using a Dionex Ultimate 3000 RSLC nanoUPLC (Thermo Fisher Scientific Inc, Waltham, MA, USA) system and a QExactive Orbitrap mass spectrometer (Thermo Fisher Scientific Inc, Waltham, MA, USA). Separation of peptides was performed by reverse-phase chromatography at a flow rate of 300 nL/min and a Thermo Scientific reverse-phase nano Easy-spray column (Thermo Scientific PepMap C18, 2 µm particle size, 100A pore size, 75 µm i.d. × 50 cm length). Peptides were loaded onto a pre-column (Thermo Scientific PepMap 100 C18, 5 µm particle size, 100A pore size, 300 µm i.d. × 5 mm length) from the Ultimate 3000 autosampler with 0.1% formic acid for 3 min at a flow rate of 10 µL/min. After this period, the column valve was switched to allow elution of peptides from the pre-column onto the analytical column. Solvent A was water +0.1% formic acid and solvent B was 80% acetonitrile, 20% water +0.1% formic acid. The linear gradient employed was 2% to 40% B in 30 min. The LC eluant was sprayed into the mass spectrometer by means of an Easy-Spray source (Thermo Fisher Scientific Inc.). All m/z values of eluting ions were measured in an Orbitrap mass analyser, set at a resolution of 70,000 and was scanned between m/z 380–1500. Data dependent scans (Top 20) were employed to automatically isolate and generate fragment ions by higher energy collisional dissociation (HCD, NCE:25%) in the HCD collision cell and measurement of the resulting fragment ions was performed in the Orbitrap analyser, set at a resolution of 17,500. Singly charged ions and ions with unassigned charge states were excluded from being selected for MS/MS, and a dynamic exclusion window of 20 s was employed. Post-run, the data was processed using Protein Discoverer (version 2.1., Thermo Scientific). Briefly, all MS/MS data were converted to mgf files, and the files were then submitted to the Mascot search algorithm (Matrix Science, London UK). The hit search was carried out against the UniProt Homo sapiens database (Homo_sapiens_20180131; 71,785 sequences; 24,195,788 residues; in-house Cambridge Proteomics, U.K.) and a common contaminant sequences database (117 sequences; 38,809 residues; in-house Cambridge Proteomics, U.K.). The peptide and fragment mass tolerances were set to 20 ppm and 0.1 Da, respectively. A significance threshold value of *p* < 0.05 and a peptide cut-off score of 20 were also applied. For the identification of putative protein–protein interaction networks for deiminated proteins identified in LN18 and LN229 GBM cells, STRING analysis (Search Tool for the Retrieval of Interacting Genes/Proteins; https://string-db.org/) was used. Protein networks were built by using the function of “search multiple proteins” in STRING, choosing “*Homo sapiens*” as the species database and applying basic settings and medium confidence, with colour lines between nodes indicating evidence-based interactions for network edges as follows: known interactions (based on curated databases, experimentally determined), predicted interactions (based on gene neighbourhood, gene fusion, gene co-occurrence) or via text mining, co-expression or protein homology. 

Human prohibitin (AAB21614.1), moesin (NP_002435.1) and STIM-1 (AAH21300.1) protein sequences were further analysed for putative disordered regions using FoldIndex© analysis (https://fold.weizmann.ac.il/fldbin/findex) [103,144].

### 4.10. Statistical Analysis

The graphs were prepared, and statistical analysis was performed using GraphPad Prism version 7 (GraphPad Software, San Diego, CA, USA). One-way ANOVA was performed followed by Tukey’s post-hoc analysis. Experiments were repeated in three biological triplicates for EV analysis and western blotting, and in three biological and three technical triplicates for microRNA analysis and cell invasion assays. NTA curves were generated by the NanoSight 3.0 software (Malvern, U.K.) with the black line representing the mean of the 5 repetitive readings per individual sample (each treatment group was repeated in 3 biological replicates) and the red line representing standard error (+/−). Histograms represent mean of data, and standard deviation (SD) is indicated by the error bars. Significant differences were considered as *p* ≤ 0.05. 

## 5. Conclusions

In summary, the findings of the current study highlight effects of PAD isozyme-specific regulation of EV release, modulation to anti-oncogenic microRNA signatures of EVs and effects on GBM cell invasion, including via effects on proteins involved in mitochondrial metabolism and invasion. Furthermore, our findings suggest differences in the regulation of KEGG pathways and protein–protein interaction networks, underlying pathogenesis of GBM tumours, via post-translational deimination. The findings presented here highlight recently identified roles for PADs in GBM, the need to assess PAD isozyme-specific processes in the heterogeneity of GBM and the potential for tailored treatment of GBM subtypes, using targeted PAD isozyme-specific inhibitors.

## Supplementary Materials

Supplementary materials can be found at https://www.mdpi.com/1422-0067/21/4/1495/s1. Supplementary Figure S1. PAD isozyme detection and deimination of PHB, STIM-1 and moesin in LN18 and LN229 GBM cells. A. PAD2 and PAD4 protein detection is relatively higher in LN18, compared to LN229 cells (by 63% and 90%, respectively), while PAD3 protein detection is relatively higher in LN229, compared to LN18 cells (by 36%). B. PHB, STIM-1 and moesin are post-translationally deiminated in both LN18 and LN229 cells as assessed by blotting the F95-enriched proteins (IP:F95) from both cell lines against the three antibodies. Relative densitometry compared to internal actin-control is represented by “R”. Supplementary Figure S2. GBM cell viability following 1 h PAD2, PAD3 and PAD4 isozyme-specific inhibitor treatment. LN18 and LN229 cell viability was assessed by PrestoBlue assay following 1 h incubation with the PAD2 (AMF30a; 5 µM), PAD3 (Cl4-amidine; 5, 10, 50 and 100 µM) and PAD4 (GSK199; 10 µM) isozyme-specific inhibitors. Supplementary Figure S3. Protein–protein interaction network analysis of deiminated proteins in LN18 cells. KEGG pathways identified for F95 enriched proteins in LN18 cells are highlighted for: (A) immune responses, (B) metabolic pathways, (C) cancer-related pathways. Supplementary Figure S4. Protein–protein network analysis of deiminated proteins in LN229 cells. KEGG pathways identified for F95 enriched proteins in LN229 cells are highlighted for: (A) immune responses, (B) metabolic pathways, (C) cancer-related pathways. Supplementary Table S1. FoldIndex© analysis of prohibitin, moesin and STIM-1. All three proteins were verified to be deiminated in LN18 and LN229 GBM cells under normal culture conditions. The number of disordered regions, residue length of the longest disordered region, total number of disordered residues, as well as number of arginines present in the total number of residues for the individual proteins is shown. Supplementary Table S2. LC-MS/MS analysis of F95 enriched proteins in LN18 cells. The full list of protein hits identified following protein immunoprecipitation using the pan-deimination F95 antibody in LN18 GBM cells is presented. Supplementary Table S3. LC-MS/MS analysis of F95 enriched proteins in LN229 cells. The full list of protein hits identified following protein immunoprecipitation using the pan-deimination F95 antibody in LN229 GBM cells is presented.

## Abbreviations

BSA	Bovine serum albumin
CD63	CD63 antigen; granulophysin; lysosomal-associated membrane protein 3
ECL	Enhanced chemiluminescence
EVs	Extracellular vesicles
F95	Pan-deimination/citrullination antibody
FBS	Foetal bovine serum
GBM	Glioblastoma multiforme
Flot-1	Flotillin-1
HIF	Hypoxia-inducible factor
kDa	Kilodalton
KEGG	Kyoto Encyclopedia of Genes and Genomes
LC-MS/MS	Liquid chromatography mass spectrometry
miR	microRNA
NTA	Nanoparticle tracking analysis
PAD	Peptidylarginine deiminase
PHB	Prohibitin
SDS-PAGE	Sodium dodecyl sulfate polyacrylamide gel electrophoresis
TBS	Tris buffered saline
TEM	Transmission electron microscopy
STIM-1	Stromal interaction molecule 1
WB	Western blotting
